# Editorial: Advances and challenges in adult-onset craniopharyngioma

**DOI:** 10.3389/fendo.2025.1755850

**Published:** 2025-12-15

**Authors:** Odelia Cooper, Andrea Glezer, Thomas Cuny

**Affiliations:** 1 Pituitary Center, Cedars-Sinai Medical Center, Los Angeles, CA, United States; 2 Neuroendocrine Unit, Division of Endocrinology and Metabolism, Hospital das Clinicas, University of Sao Paulo Medical School, Sao Paulo, Brazil; 3 Department of Endocrinology, Centre de Reference Maladies Rares HYPO (CRMR HYPO), U1409, Hôpital de La Conception, Marseille, France

**Keywords:** BRAF and MEK inhibitors, craniopharyngioma, hypopituitarism, hypothalamic dysfunction, methylation

Among the skull base tumors, craniopharyngiomas (CPs) are one of the most challenging to treat due to their location near, within, or surrounding the pituitary gland and stalk, and their propensity to induce pituitary hormone deficiencies. Although guidelines on the diagnosis and management have been recently published (1–3), areas of uncertainty persist, particularly for adult-onset CP. This Research Topic entitled “*Advances and Challenges in Adult-Onset Craniopharyngiomas*” provides an overview of unique and innovative research into CPs through original articles, a comprehensive review, and illustrative clinical cases. Taken together, they lay the groundwork for new research and clinical paradigms in the management of adult-onset CP.

Adamantinomatous (ACP) and papillary (PCP) subtypes are entirely distinct entities, differing in their histological, genetic, and clinical behavior. In a large retrospective study, Guo et al. compared pituitary function after neurosurgery between ACPs and PCPs. Interestingly, although the PCP group presented with more suprasellar tumor extension, mass effects, and arginine vasopressin (AVP) deficiency before surgery, the ACP group more commonly presented with adrenocorticotropin and growth hormone (GH) deficiencies. Most prior studies evaluating neuroendocrine dysfunction in patients with ACP and PCP showed that the ACP subtype was associated with a high recurrence rate and poorer prognosis (4–6), although some pointed to a worse prognosis in the PCP group (7, 8). The current study’s strong points are its large cohort, which has good representation of both CP subtypes; the similarity in tumor volume and radiotherapy use in both groups; and the evaluation of pituitary dysfunction severity, performed by measuring the cumulative number of adenohypophyseal and neurohypophyseal hormone deficiencies. Although ACP is more commonly located in the subdiaphragmatic region and PCP in the third ventricle, ACP is usually more infiltrative and adherent to neurovascular structures, which could explain its worse prognosis (9). This study supports the different outcomes in neuroendocrine dysfunction between the two subtypes of CP and reinforces the importance of treating them differently.

Understanding of molecular mechanisms underlying ACP and PCP has considerably evolved over the years. Description of somatic mutations in the *CTNNB1* and *BRAF* genes, respectively, endorses a theranostic approach given the dramatic antitumoral effects with BRAF and MEK inhibitors in patients with PCP (10). However, less is known about the molecular events that might drive a higher risk of relapse. Marrero-Gutiérrez et al. conducted methylomic and transcriptomic analyses in ACP samples to explore the potential interplay between DNA methylation and RNA expression signatures for diagnostic and prognostic applications. They found a dichotomic molecular pattern of ACP made by two clusters of tumors, ACP-A and ACP-B. Clinical and histopathological characteristics were similar between clusters, and there were no differences in the frequency of tumor progression (62% and 80%, respectively, for ACP-A and ACP-B, *P*>0.9), nor in the proportion of patients undergoing more than one surgical procedure (55 vs 67%, *P* = 0.67) or adjuvant radiotherapy (56% vs 33%, *P* = 0.61). However, they observed a trend for a longer median progression-free survival in patients with cluster ACP-A than with ACP-B (140.1 vs 25.1 months; *P* = 0.1). Functional enrichment analysis highlighted differential expression and, therefore, key roles of genes involved in synaptic modulation, nervous system development, and cell adhesion, as well as pathways linked to RAS signaling, GTPase activity, and membrane potential regulation. Work from the same group previously identified two similar clusters in the methylome of ACP using unsupervised methods (11). In that study, the hypomethylated subgroup was associated with a higher rate of *CTNNB1* mutations and an increased tumor size. Together, these works suggest that a comprehensive molecular approach toward CPs is likely critical to personalized and integrative clinical management.

The need for advanced -*omics* technologies in the study of CP is further underscored in the comprehensive review from de Oliveiro Neto et al. This work highlights the molecular homogeneity of PCP compared with ACP, with somatic *BRAF_V600E_* mutations identified in virtually all cases. Accordingly, application of BRAF and MEK inhibitors in this setting has led to durable partial responses in more than 90% of patients with PCP (10, 12). By contrast, ACPs, despite carrying somatic mutations of *CTNNB1* in the majority of cases, are characterized by a complex interplay of several molecular and cellular actors involving both the tumor and its microenvironment beyond *CTNNB1* mutations and β-catenin aberrant nucleocytoplasmic accumulation. The review summarizes the efficacy of molecularly targeted therapy agents used in ACP, many of which are tested in small cohorts of patients or even isolated case reports. The review also emphasizes the need for integrated analyses encompassing genomic, transcriptomic, proteomic, and spatial data, combined with detailed microenvironment characterization in this subset of CP. The last section of the review provides an overview of the inflammatory mediators and immune response components involved in the pathophysiology of ACP, with a goal of helping the reader appreciate the rationale of ongoing clinical trials in this area.

Meyer et al. presents two cases that illustrate the specific features, challenges, and therapeutic opportunities for patients with adult-onset CP. In reviewing the literature, the authors distinguish the clinical picture of CP in children vs in adults, noting that nausea/vomiting, photophobia, AVP deficiency, and GH deficiency are most commonly found in children, whereas in adults the most common symptom is insidious visual worsening (13). They further note that although differentiation between ACP and PCP is pathologic and genetic, radiologic features such as calcification on CT and T1 hyperintensity on MRI suggest ACP (14). The authors extensively reviewed the results of primary surgical studies conducted over the last 15 years, showing that recurrence is lower in cases with gross total resection. Although mortality is increased in patients with CP, especially due to cardiovascular disease (15), systematic analysis of mortality and morbidity in adults is limited, as most studies evaluated outcomes in pediatric patients or a mixed pediatric-adult population. Morbidity results not only from visual deficiencies and hypopituitarism, but also from other chronic complications such as obesity and neurocognitive impairments that severely impair quality of life. The authors thus emphasize the need for a specialized multidisciplinary team in the management of adult-onset CP.

Finally, Biswas et al. provide a mutation-guided approach to the treatment of patients with PCP. They present two patients with PCP in which the tumor and/or cyst wall were inseparable from the hypothalamus, precluding extensive resection. As the pathology confirmed the mutation in BRAF_V600E_, both patients were treated postoperatively with BRAF-MEK inhibitors (10) and showed sustained reduction in tumor size. Preservation of the pituitary stalk and hypothalamic tissue is an important goal for CP surgery as evidence of the burden of hypothalamic dysfunction from surgical trauma to the hypothalamic apparatus is increasingly recognized (16–19). Approximately 50% of patients with CP develop hypothalamic obesity (20), and more extensive damage to the hypothalamus further increases the risk (21). Guiding treatment based on mutation status opens new options of medical treatment for PCP and may reframe our traditional approach to this tumor.

This collection of five articles illustrates the complexities of caring for patients with adult-onset CP. They address key gaps in our understanding of tumor biology and the effect of these tumors on endocrine and hypothalamic dysfunction. By striving to understand the molecular mechanisms underlying these tumors, we can identify targeted treatments to minimize the long-term complications. Furthermore, these studies impress upon us that management of these patients by a multidisciplinary team approach in a pituitary center of excellence is vital to optimizing care. It is our hope that this Research Topic will stimulate further research into this rare tumor.
